# Oncologists’ beliefs about people with psychotic disorders : a qualitative study

**DOI:** 10.1192/j.eurpsy.2022.1697

**Published:** 2022-09-01

**Authors:** A. Le Glaz, C. Lemey, M. Walter, C. Lemogne, C. Flahault

**Affiliations:** 1 Brest Medical University Hospital, Psychiatry, Bohars, France; 2 AP-HP, Assistance Publique - Hôpitaux de Paris, Adult Psychiatry, Paris, France; 3 Université de Paris – INSTITUT DE PSYCHOLOGIE, Laboratoire De Psychopathologie Et Processus De Santé (ur 4057), Boulogne Billancourt, France

**Keywords:** Oncologists, beliefs and attitudes, PSYCHOTIC DISORDERS, stigma

## Abstract

**Introduction:**

Cancer is the second major cause of death among people with psychotic disorders. With the same incidence, mortality in these patients remains higher than in the general population. As stigma has been identified as a risk factor for excess mortality, we focused on oncologists’ beliefs and attitudes towards people with psychotic disorders.

**Objectives:**

The aim of this study was to uncover physicians’ representations about the impact of psychosis on oncological care.

**Methods:**

In this qualitative study, individual semi-structured interviews were conducted with 20 physicians working in oncology in the University Hospital of Brest (France). Transcribed interviews were thematically analyzed. This study meets the COREQ criteria.

**Results:**

Psychosis is described as a broad-spectrum condition whose severity ranges from the “mild” patient with imperceptible abnormality to the “severe” patient with cognitive and affective deficits. Oncologists identified behavioral and emotional symptoms which may modify the patient-physician relationship with difficulties to interact. Some of them consider that these patients are not interested in their health and will not get involved in oncological care. While the psychotic disorder is not considered as a limiting factor per se, oncologists felt concerned about being stigmatizing. They mentioned different aspects (like anticipation of non-compliance or inability to get help) that lead to changes in conventional treatment regimens and may result in a loss of opportunity.

**Conclusions:**

Oncologists’ beliefs may lead to stigmatizing attitudes towards people with psychotic disorders who may not be given the best possible chances. Thus, these specific elements should be the basis for collaboration between psychiatrists and oncologists.

**Disclosure:**

No significant relationships.

